# Social inequalities in children’s oral health-related quality of life: the Generation R Study

**DOI:** 10.1007/s11136-017-1679-1

**Published:** 2017-08-18

**Authors:** Lea Kragt, Eppo B. Wolvius, Hein Raat, Vincent W. V. Jaddoe, Edwin M. Ongkosuwito

**Affiliations:** 1 Department of Oral and Maxillofacial Surgery, Special Dental Care and Orthodontics, Erasmus University Medical Centre, P.O Box 2040, 3000CA Rotterdam, The Netherlands; 2 Department of Public Health, Erasmus University Medical Centre, Rotterdam, The Netherlands; 3 Department of Pediatrics, Erasmus University Medical Centre, Rotterdam, The Netherlands; 4 Department of Epidemiology, Erasmus University Medical Centre, Rotterdam, The Netherlands; 5 The Generation R Study Group, Erasmus University Medical Centre, Rotterdam, The Netherlands

**Keywords:** Quality of life, Oral health, Social inequalities, Children

## Abstract

**Purpose:**

Oral health-related quality of life (OHRQoL) is the most important patient-reported outcome measure in oral health research. The purpose of the present research was to study the association of family socioeconomic position (SEP) with children’s OHRQoL.

**Methods:**

This cross-sectional study was embedded in the Generation R Study, a population-based cohort study conducted in Rotterdam, The Netherlands. For the present study, OHRQoL was assessed of 3871 ten-year old children. Family SEP was assessed with the following indicators: maternal/paternal education level, maternal/paternal employment status, household income, benefit dependency, and family composition. Linear regression analyses were performed to evaluate the (independent) associations of family SEP indicators with OHRQoL.

**Results:**

The median (90% range) OHRQoL score of the participating children was relatively high [50.0 (43.0–53.0)]; however, OHRQoL was consistently lower in children with low family SEP. Positive associations were found for all SEP indicators (*p*-values <0.05) except maternal employment status and family composition. Benefit dependency, paternal employment, and household income were the most strongly associated with OHRQoL. No family SEP indicator was significantly associated with OHRQoL independent of the other indicators.

**Conclusions:**

Based on the present findings, interventions and policies promoting good oral health and oral well-being should target children from low socioeconomic position. More research is needed, however, to understand the pathways of social inequalities in children’s OHRQoL especially for the effects of material resources on subjective oral health measures.

**Electronic supplementary material:**

The online version of this article (doi:10.1007/s11136-017-1679-1) contains supplementary material, which is available to authorized users.

## Background

Oral health-related quality of life (OHRQoL) is a commonly used patient-reported outcome measures in dental research. This measure is designed to assess the impact of oral diseases from the patient perspective and is thus subjective and multidimensional. It is particularly suited to assess oral health of individuals, because it encompasses their physical function, psychological state, social interaction, and somatic sensation [1]. Quality of life measures in medical and dental research become increasingly important because of the patients’ more active participation in their health treatment, because of the need for new evidence in oral health practices, and because more and more diseases cannot be cured by the treatment although they improve the patient’s condition [1, 2].

Research on OHRQoL is directed by the Wilson and Cleary model, which shows the possible link between biological and clinical variables, characteristics of the individual and the environment and other nonmedical factors on OHRQoL [3, 4]. Many studies suggest that socioeconomically deprived persons tend to have worse oral health and unmet dental treatment needs [5–9]. In line with the Wilson and Cleary model, these inequalities have also been shown in subjective oral health measures among adolescents, adults, and the elderly [10–13]. Also several studies have been conducted on the relation between socioeconomic status and children’s OHRQoL, but these make use of various study populations, various socioeconomic indicators, various methods, and statistical analyses [13]. Yet, the evidence for the relation between family socioeconomic position (SEP) and OHRQoL in the preschool-aged and the school-aged children is inconclusive [13, 14].

OHRQoL is important and it is necessary to identify risk groups at an early stage, because poor oral health will track through childhood into adolescence and adulthood [15–17]. Therefore, the aim of the present study was to investigate the associations of family SEP with children’s OHRQoL. For this research, we used the data from the Generation R Study, which is a large multiethnic birth cohort in Rotterdam, The Netherlands.

## Material and methods

### Study design

This cross-sectional study is performed within the Generation R Study, which is an ongoing multiethnic population-based prospective cohort study. This study has been described in detail elsewhere [18]. The Generation R Study has been conducted following the World Medical Association Declaration of Helsinki and was approved by the Medical Ethics Committee at Erasmus Medical Centre, Rotterdam, The Netherlands (MEC-2012-165). Written consent was obtained from all participants before data collection was started.

### Study population

Invitations to participate in the study were given to all pregnant women with an expected delivery date between April 2002 and January 2006 living in the study area (Rotterdam, The Netherlands). From the original 9749 live-born children included in the Generation R cohort, 7393 children still participated in the follow-up period from the children’s age of 9 years onward. From these, we selected the children with available data on OHRQoL, which was assessed at the median (90% range) age of 9.79 (9.48–10.47). In total, 3871 children were included in this study.

### OHRQoL

OHRQoL of the children was assessed by parental questionnaires. For this, a Dutch 11-item version of the Children’s Oral Health Impact Profile (COHIP) was used, which has previously been validated in a comparable population [19]. The questions cover the five subdomains of children’s oral health: oral symptoms, functional well-being, emotional well-being, school, and peer interaction. The questions inquire about the frequency of oral health impacts on daily life and are answered on a 5-point Likert scale: never (5 points), almost never (4 points), sometimes (3 points), often (2 points), and always (1 point). All answers were added up to a final OHRQoL score (range 11–55 points), with the highest score indicating the best quality of life. Missing values in the responses to the questionnaire were replaced by the personal mean score of the remaining answers to the questions, as it is proposed by other researchers who used the original version of the COHIP [20]. If there were more than 30% of the answers missing, the participant was excluded from the analysis.

### Family social position

Following socioeconomic indicators of family SEP were considered in the present study: maternal and paternal education level, maternal and paternal employment status, and net household income, which are all traditional family SEP indicators [21]. We also used receiving benefits and single parenting as additional family SEP indicators, because these were associated with oral health in previous research [13, 22]. Parental education was assessed at the children’s age of six by questionnaires and defined as low (no education, primary school, lower or intermediate vocational training, general school, or first year of higher vocational training) or high (higher vocational training, university, or PhD degree). Also, information on paternal and maternal employment status was assessed by questionnaires at the children’s age of six and categorized into no paid job (unemployed, disabled, welfare recipient, housewife or student, or other nonpaid work) or paid job (paid or self-employed). In addition, information on net household income (≤2000€ vs. >2000€), receiving benefits (no vs. social security, unemployment benefits, disability allowances, or other), and single parenting was assessed in parental questionnaires around children’s age of ten, which were the same as for the assessment of OHRQoL.

### Confounders

Based on the literature and prior experience in clinical practice, child’s sex, age, and ethnic background were considered confounders in the association between family SEP and OHRQoL. In addition, the following oral health variables were considered as potential confounders for the relationship between family SEP and OHRQoL: caries experience, orthodontic treatment need based on either the Dental health component (IOTN-DHC) or the aesthetic component (IOTN-AC) of the Index of Orthodontic treatment need and self-perceived orthodontic treatment need [23]. Because not all the considered confounders were assessed in the same follow-up period as OHRQoL, i.e., around the children’s age of 10, we used some measurements from previous time points.

Child’s ethnicity was defined following the guidelines for classification by Statistics Netherland [24]. Children’s ethnic background, assessed at enrollment in the Generation R Study, was based on the country of birth of the parents. Children of parents, with both parents being born in the Netherlands, were classified as native Dutch. If at least one of the parents was not born in the Netherlands, the child was classified as nonnative Dutch.

Caries experience was assessed at the children’s age of six with the decayed missing and filled teeth index (dmft) which ranges from 0 to 20. The dmft-score (decayed missing and filled teeth-score) of each child was obtained from intraoral photographs. Before taking photographs, the children brushed their teeth, and the teeth were dried with a cotton roll. The images were taken with one of the two intraoral cameras: the Poscam USB intraoral (Digital Leader PointNix) and Sopro 717 (Acteon) autofocus camera. Both cameras had a resolution of 640 × 480 pixels and a minimal scene illumination of f 1.4 and 30 lx. The whole dentition was captured with 10 photographs. The photographs were judged by one pediatric dentist (intrarater reliability *Κ* = 0.95), and a second calibrated pediatric dentist judged 10% of the photographs (interrater reliability of *Κ* = 0.62). Scoring dental caries per tooth on intraoral photographs has been described elsewhere with a high sensitivity (85.5%) and specificity (83.6%) compared with ordinary oral examination [25]. If one or more primary teeth were not able to be judged on the photographs, no dmft-score was given. Children were categorized into no caries experience (dmft = 0) vs. caries experience (dmft > 0).

The IOTN-DHC and IOTN-AC were assessed from photographic and radiographic records of the children taken around the age of ten. Assessment of the IOTN on a combination of photographic and radiographic records has been validated previously [26]. After 6 months, 10% of the photographs were reassessed first by the same examiner (LK) and second by another examiner (EO). With these measurements, intrarater reliability (linear-weighted *Κ* = 0.84) and interrater reliability were calculated (linear-weighted *Κ* = 0.68).

Self-perceived orthodontic treatment need was measured in the parental questionnaires around the children’s age of ten with the question: “Do you want your child to get braces?” The question was answered on a five-point Likert scale, with the answer possibilities ranging from ‘strongly disagree’ to ‘strongly agree.’ Answers were categorized into ‘self-perceived need’ (strongly/somewhat agree), ‘borderline self-perceived need’ (do not agree or disagree), and ‘no need’ (strongly/somewhat disagree).

### Statistical analysis

First, we used descriptive statistics to characterize the study population.

The associations of family SEP indicators with OHRQoL were analyzed with series of weighted least squares linear regression models. Different regressions models were run, having each of the SEP variables as an independent variable. Finally for all indicators, three different models were created. First, we created the crude model adjusted for child’s age, gender, and ethnic background only. Second, we created model 1 adjusted for confounders. Potential confounders were included in model 1: when they changed the estimate by approx. 10%, or when they were significant when entered into the crude model, or when the *R* ^2^ of the model improved. Finally, we created model 2 adjusted for confounding variables and all other family SEP indicators simultaneously. Model 2 was created to evaluate the independent effects of each of the SEP indicators. To assess the explanatory effects of the oral health variables on the association of a particular SEP indicator with OHRQoL, the differences between the crude model and model 1 were compared:

$$ \left[ {\left( {(\beta_{{{\text{crude}}{\kern 1pt} {\text{\, model}}}} - \beta_{{{\text{\, model}}{\kern 1pt} 1}} )/\beta_{{{\text{crude}}{\kern 1pt} {\text{model}}}} ) \times 100\% } \right)} \right]. $$

This approach allows one also to evaluate the influence of SEP indicators on oral health from the patient perspective. Likewise, the explanatory effects of the other family SEP indicators on the association between a particular SEP indicator with OHRQoL were assessed. Significance of the difference was assessed with a test for heterogeneity. In addition, we categorized OHRQoL based on the median value and conducted logistic regression models analogous to the linear regression models.

Finally, we tested for multicollinearity in model 2 by obtaining the tolerance and VIF values for each determinant and covariate. Tolerance values above 0.10 and VIF values below 10 were considered acceptable to rule out multicollinearity [27, 28].

We conducted a nonresponse analysis by comparing children with data available on OHRQoL with the children that had no data on OHRQoL on all family SEP indicators and confounders using Mann–Whitney-*U* tests or Chi-Square tests. Missing data in the determinants and covariates were multiple imputed based on the other determinants, covariates, and OHRQoL. Ten imputed datasets were created using a fully specified model for which we present the pooled regression coefficients with 95% confidence intervals (aβ, 95% CI).

All analyses were performed using IBM SPSS Statistics version 21.0 for Windows (SPSS INC., Chicago, IL, USA). A significance level of *p* < 0.05 was used for all analysis.

## Results

### Sample characteristics

The nonresponse analysis showed that children without information on OHRQoL had more often parents from a lower socioeconomic status (supplemental Table S1). In Table 1, the characteristics of the study population are presented. Most of the children were native Dutch (67.8%). Approximately one-third of all the children had a mother or father with a low education level (32.7, 31.5% resp.). Almost one-fifth of the children lived in a household with an income below 2000€ per month. Prevalence of oral health variables were relatively high, with approximately 18.4% of the children having caries experiences, 29.6% of the children having objective, and 46.5% having subjective orthodontic treatment need. The median (90% range) OHRQoL score of the children was 50.00 (43.00–53.00).

**Table 1:** Sample characteristics of the study population (*n* = 3871)

	Total *n* (%)	Missing *n* (%)
Maternal education level^a,c^		
Low	1267 (32.7)	254 (6.6)
High	2350 (60.7)	
Paternal education level^a,c^		
Low	1220 (31.5)	489 (12.6)
High	2162 (55.9)	
Maternal employment status^c^		
Paid job	2785 (71.9)	414 (10.7)
No paid job	672 (17.4)	
Paternal employment status^c^		
Paid job	3164 (81.7)	558 (14.4)
No paid job	149 (3.8)	
Household income^d^		
<2000€	643 (16.6)	273 (7.1)
>2000€	2955 (76.3)	
Receiving benefits^b,d^		
Yes	394 (10.2)	117 (3.0)
No	3360 (86.8)	
Family composition^d^		
One parent	553 (14.3)	126 (3.3)
Two parents	3192 (82.5)	
Ethnicity		
Native Dutch	2626 (67.8)	61 (1.6)
Non-Dutch	1184 (30.6)	
Childs sex		
Male	1923 (49.7)	0 (0.0)
Female	1948 (50.3)	
Childs age		
Median (90% range)	9.79 (9.49–10.47)	0 (0.0)
Caries experience^c^		
No	2167 (56.0)	991 (25.6)
Yes	713 (18.4)	
Orthodontic need^d^		
No	1902 (49.1)	823 (21.3)
Yes	1146 (29.6)	
Aesthetic orthodontic need^d^		
No	1691 (43.7)	927 (23.9)
Borderline	1006 (26.0)	
Definite	247 (6.4)	
Self-perceived orthodontic need^d^		
No	1075 (27.6)	22 (0.6)
Borderline	980 (25.2)	
Yes	1794 (46.5)	
OHRQOL^d^		
Median (90% range)	50.00 (43.00–53.00)	0 (0.0)

The table is based on the nonimputed dataset. Data are presented as absolute numbers with percentages for categorical data and as median with 90% range for continuous data

^a^ Educational level: low = no education, primary school, vocational training, general secondary school, and first-year higher vocational training; high = higher vocational training, university, or PhD degree

^b^ Benefits: social security, unemployment benefits, disability allowances, and other

^c^ Assessed at children’s age of 6

^d^ Assessed at children’s age of 10

### Association of family SEP indicators with OHRQoL

The correlation between all family SEP indicators varied between 0.08 and 0.54 (supplemental Table S2). The VIF values for determinants and confounders in the models used to describe the associations between family SEP indicators and OHRQoL were all less than 1.5, and the tolerance values were all above 0.70 (supplemental Table S3). The results of the regression analysis having each of the SEP variables individually as an independent variable are presented in the supplement (supplemental Table S4). In Table 2, the associations between family SEP and OHRQoL are presented. Children of fathers and mothers with low educational level had lower OHRQoL than children of fathers with a high education level [crude model father: aβ: −0.45 (95% CI: −0.68 to −0.22); crude model mother: aβ: −0.34 (95% CI: −0.56 to −0.11)]. Similarly, significantly lower OHRQoL was seen in children of unemployed fathers [aβ: −0.81 (95% CI: −1.39 to −0.22)], children with a low household income [aβ: −0.67 (95% CI: −0.99 to −0.36)], children living in a household that receives any kind of benefits [aβ: −0.68 (95% CI:−1.07 to −0.30)], or a single-parent family [aβ: −0.54 (95% CI: −0.87 to −0.22)]. Thus, all family SEP indicators, except maternal educational level, were negatively associated with OHRQoL.

**Table 2:** Associations of socioeconomic indicators with OHRQOL at children’s age of 10

	Crude model	Model 1	Model 2
	*β* [95% CI]	*β* [95% CI]	*β* [95% CI]
Maternal education level^a, c^			
Low	**−0.34 [−0.56 to −0.11]**	**−0.22 [−0.44 to −0.01]**	0.03 [**−**0.21 to 0.27]
Paternal education level^a, c^			
Low	−**0.45 [−0.68 to −0.22]**	**-0.35 [−0.55 to −0.14]**	**−**0.20 [**−**0.44 to 0.03]
Maternal employment status^c^			
No paid job	**−**0.24 [**−**0.54 to 0.06]	0.11 [−0.38 to 0.17]	0.11 [−0.18 to 0.40]
Paternal employment status^c^			
No paid job	−**0.81 [**−**1.39 to** −**0.22]**	−**0.64 [**−**1.18 to** −**0.11]**	−0.29 [-0.84 to 0.26]
Household income^d^			
<2000€	−**0.67 [**−**0.99 to** −**0.36]**	−**0.59 [**−**0.88 to** −**0.31]**	−0.21 [−0.57 to 0.16]
Receiving benefits^b, d^			
Yes	−**0.68 [**−**1.07 to** −**0.30]**	−**0.64 [**−**0.99 to** −**0.28]**	−0.33 [−0.70 to 0.04]
Family composition^d^			
Single parent	−**0.54 [**−**0.87 to** −**0.22]**	−**0.50 [**−**0.80 to** −**0.20]**	−0.09 [−0.42 to 0.24]

The data are presented as linear regression coefficients (*β*) with 95%-confidence intervals (95% CI). The crude model is adjusted for gender, age, and ethnicity only. Model 1 is additionally adjusted for confounders: caries experiences, orthodontic treatment need, aesthetic treatment need, and self-perceived orthodontic treatment need. Model 2 is additionally adjusted for confounders and the other socioeconomic factors

Significant associations are printed bold

^a^ Educational level: low = no education, primary school, vocational training, general secondary school, and first-year higher vocational training; high = higher vocational training, university, or PhD degree

^b^ Benefits: social security, unemployment benefits, disability allowances, and other

^c^ Assessed at children’s age of 6

^d^ Assessed at children’s age of 10

All of these associations remained significant after adjustment for oral health variables (model 1, Table 2). The oral health variables explained between 1.9 and 47.6% of the relationship between the different SEP indicators and OHRQoL. After adjustment for the other family SEP indicators, benefit dependency and paternal employment status were the most strongly associated with OHRQoL [aβ: −0.33 (95% CI: −0.70 to −0.04), resp. aβ: −0.29 (95% CI: −0.84 to −0.26)]. However, there were no significant independent family SEP associations with OHRQoL found (model 2, Table 2). The associations between family SEP indicators and OHRQoL were explained by the other family SEP indicators between 16.7 and 75.0%. The results based on the logistic regression models are comparable with the linear regression analysis and presented in the supplement (supplemental Table S5).

## Discussion and conclusion

Family SEP was consistently positively associated with OHRQoL. Moreover, children with lower family SEP perceived lower OHRQoL independent of their objective oral health status.

Our results suggest that not only clinical variables, such as caries and malocclusions, are associated with lower OHRQoL, but also different socioeconomic and environmental variables interfere significantly in children’s conditions of daily life. This is in line with other research that showed how socioenvironmental factors are related to lower OHRQoL in the 12-year old children [14], as well as with studies that show socioeconomic inequalities in objective oral health [7, 29].

Many studies suggest that, but have not found conclusive evidence, the association of family SEP with children’s OHRQoL may be related to oral health behavior, like tooth brushing frequency, sugar intake, and regular dental visits [30–32]. However, in different studies, low socioeconomic status was associated with less oral hygiene, higher added sugar intake, and less dental service use [33–35]. We did not specifically adjust our models for oral health behavior. Remarkably, our results stay significant after adjustment for oral health variables like caries experience and orthodontic treatment need. Thus, children with a low family SEP do perceive lower oral health, although it might not necessarily be true. This indicates that the effect of family SEP on OHRQoL is attributed to several additional factors, the so-called mediators, rather than simply to oral hygiene and oral health.

One mediator that might contribute to the association between family SEP with OHRQoL is related to aspects of self-esteem and self-perception about oral health and body image. One study showed that socioeconomic disparities in self-perceived oral health might partly be mediated by psychosocial factors like self-esteem [36]. Other literature studies about the influence of self-esteem and (self-perceived) oral health are mainly focused on the orthodontic field and remains inconclusive [37, 38]. Unfortunately, studies on the relationship between family SEP and self-esteem are scarce. Whereas self-esteem has been shown to be significantly associated with quality of life, the associations between family SEP indicators and self-esteem appear inconsistent [39, 40]. Because we did not include self-esteem in our analysis, we cannot conclude about its role in the association between family SEP and OHRQoL. However, considering the Wilson and Cleary model of (oral health-related) quality of life [3], we highly recommend further research to understand the role of self-esteem in relationship with environmental factors and OHRQoL.

The strength of the associations between family SEP indicators and OHRQoL slightly varied. In general, family SEP indicators are associated with each other [21, 22]. This lack of independence among these variables makes it difficult to conclude which factor is most important. Maternal education and employment status were less related to children’s OHRQoL. One reason for this might be that the father is still most often the principal earner of the family, which would make maternal variables less appropriate as SEP indicators. Indeed, the correlation between maternal and paternal education and employment status was fairly low (supplemental Table S2). These considerations suggest that maternal employment status might not be used as favorable socioeconomic indicator in oral health research among populations comparable to the Generation R Cohort. The family SEP indicators directly related to material resources of the household (benefit dependency, paternal employment status, and household income) were the most strongly related to OHRQoL (see also supplemental Table S4). Dental treatment and care often involves high costs. As this might indicate that oral health care is less accessible to children with lower family socioeconomic position, this finding should alert oral health care providers and policy makers.

There are several important theoretical reasons why it is important to study family SEP with children’s OHRQoL. Family SEP refers to the social and economic factors influencing which position individuals have within the society [21]. A low SEP has a negative influence on adult’s oral health, and parental oral health in turn is a strong predictor for child oral health [41]. In addition, the influences of SEP act over the life course and are therefore important to study as early as possible. Reduced child oral health is a strong predictor for impaired oral health in later life [42]. Finally, all the different influences on OHRQoL help to understand why relationships between clinical status and OHRQoL are sometimes weak and inconsistent.

The social inequalities in children’s OHRQoL found in the present study indicate that policies and interventions aimed to promote oral health behaviors and prevent oral disease as well as discomfort among socially deprived are highly warranted. Based on our study, these strategies should take social disadvantage into account along with the other mediating factors such as oral health behaviors, cultural differences, or self-esteem and could involve education, social benefits for dental treatments, or introducing insurance covering various kinds of dental treatments.

Certainly, some limitations of the present study need to be discussed. First, as in every observational study, our results might be affected by residual confounding, although we have constructed the fully adjusted models to assess the independent effects of different family SEP indicators. Yet, family SEP is a complex concept, and we did not include all kinds of family SEP indicators, as, for example, wealth or neighborhood SEP indices [21, 22]. Second, in this study, children’s’ OHRQoL was assessed by parental questionnaires which might have introduced information bias. We used parental reports because of practical reasons and because several studies found parents to be good proxies for children’s OHRQoL [43–45]. Third, another potential source of information bias in the present study might be due to the assessment of certain SEP indicators at earlier time points. Certain SEP indicators are dynamic and change over time. However, educational level, for example, is known to be relatively stable [21]. Moreover, we found consistent associations for almost all SEP indicators with OHRQOL, which suggest that the earlier assessed data are good proxies for the current SEP. Fourth, with regard to the original sample size of the Generation R study, this study had a great number of losses to follow-up. This could have resulted in a selection bias, if the association between family SEP and OHRQoL would be different between the excluded and included study population. However, this seems unlikely. Fifth, because the population of the Generation R Study has a generally high SEP, the generalizability of our results are potentially limited. Sixth, because we analyzed many different socioeconomic indicators in this study, multiple testing might be seen as a threat to this study. However, because in our opinion testing all these different indicators fit into one single hypothesis, i.e., a consistent relationship between family SEP and OHRQoL, we did not adjust for a multiple testing problem. Last, the various family SEP indicators could be seen as mediators for the association between socioeconomic indicators and OHRQoL. Therefore, a few of the effects of these indicators are not confounded but caused by other socioeconomic indicators. This is most easily explained for family composition, as single households, for example, are linked to lower household income or benefit dependency [21, 22, 46]. We saw in our analysis that other socioeconomic indicators explain 75.0% of the association between family composition and OHRQoL. As a consequence, relationships between the various socioeconomic indicators with OHRQoL may have been underestimated in model 2. Still, model 2 was not affected by multicollinearity, as tolerance and VIF values for all covariates were within the accepted range.

The advantages of this study include the large and ethnically diverse study population and the availability of multiple indicators of family SEP. A post hoc statistical power calculation indicated that a sample of 910 children would have been sufficient to show the present findings with 80% statistical power (based on number of predictors =14, *α* = 0.05, lowest *R* ^2^ tested = 0.02) [47]. This study, however, includes 3871 children. To our knowledge, this is the first study that investigates the association between family SEP and OHRQoL in such a large multiethnic cohort.

In conclusion, family SEP was consistently associated with OHRQoL, as indicated by children from low family SEP having lower OHRQoL. These associations were independent of their clinical oral health status, reinforcing the importance of OHRQoL as outcome measure in oral health research. Given these disparities, interventions and policies promoting good oral health and oral wellbeing should target children from low socioeconomic position. Nevertheless, more research is needed to understand the pathways of social inequalities in children’s OHRQoL.

## Electronic supplementary material

Below is the link to the electronic supplementary material.

Supplementary material 1 (DOCX 31 kb)
