# Supplementary material for: Social inequalities in children’s oral health-related quality of life: the Generation R Study
Source: Qual Life Res. 2017 Aug 18;26(12):3429–37. doi: 10.1007/s11136-017-1679-1 (PMC5681972; doi:10.1007/s11136-017-1679-1)
Supplement: Supplementary file 1 — Supplementary material 1 (DOCX 31 kb) [file 11136_2017_1679_MOESM1_ESM.docx]

**Appendix**

| **Table S1** Non response analysis (n = 8548) | | | |
| --- | --- | --- | --- |
|  | Included (n = 3871) | Excluded (n = 4677) | p-value |
| Maternal education level­­ ^a, c^ |  |  |  |
| low | 1267 (35.0) | 1369 (54.4) |  |
| high | 2350 (65.0) | 1148 (45.6) | < 0.001 |
| Paternal education level ^a, c^ |  |  |  |
| low | 1220 (36.1) | 1140 (51.9) |  |
| high | 2162 (63.9) | 1057 (48.1) | < 0.001 |
| Maternal employment status ^c^ |  |  |  |
| paid job | 2785 (80.6) | 1584 (67.6) |  |
| no paid job | 672 (19.4) | 760 (32.4) | < 0.001 |
| Paternal employment status ^c^ |  |  |  |
| paid job | 3164 (95.5) | 1964 (92.0) |  |
| no paid job | 149 (4.5) | 171 (8.0) | < 0.001 |
| Household income^d^ |  |  |  |
| < 2000€ | 643 (17.9) | 372 (31.7) |  |
| > 2000€ | 2955 (82.1) | 800 (68.3) | < 0.001 |
| Receiving benefits ^b, d^ |  |  |  |
| Yes | 394 (10.5) | 201 (16.3) |  |
| No | 3360 (89.5) | 1032 (83.7) | < 0.001 |
| Family composition ^d^ |  |  |  |
| One parent | 553 (14.8) | 282 (23.1) |  |
| Two parents | 3192 (85.2) | 940 (76.9) | < 0.001 |
| Ethnicity |  |  |  |
| native Dutch | 2626 (68.9) | 1938 (46.3) |  |
| non- Dutch | 1184 (31.1) | 2251 (53.7) | < 0.001 |
| Childs sex |  |  |  |
| male | 1923 (49.7) | 2416 (51.7) |  |
| female | 1948 (50.3) | 2260 (48.3) | 0.068 |
| Childs age |  |  |  |
| median (90% range) | 9.79 (9.48-10.47) | 9.98 (9.56-11.18) | < 0.001 |
| Caries experience ^c^ |  |  |  |
| no | 2167 (75.2) | 1430 (60.7) |  |
| yes | 713 (24.8) | 925 (39.3) | < 0.001 |
| Orthodontic need ^d^ |  |  |  |
| no | 1902 (62.4) | 985 (60.2) |  |
| yes | 1146 (37.6) | 652 (39.8) | 0.139 |
| Aesthetic need |  |  |  |
| no | 1691 (57.4) | 908 (57.5) |  |
| borderline | 1006 (34.2) | 535 (33.9) |  |
| yes | 247 (8.4) | 136 (8.6) | 0.958 |
| Self-perceived orthodontic need ^d^ |  |  |  |
| no | 1075 (27.9) | 1 (9.1) |  |
| borderline | 980 (25.5) | 2 (18.2) |  |
| yes | 1794 (46.6) | 8 (72.7) | 0.199 |
| Data are presented as absolute numbers with percentages for categorical data and as median with 90% range for continuous data. P-values are based on chi-square tests and Mann-Whitney-U tests. ^a^ educational level: low = no education, primary school, vocational training, general secondary school and first year higher vocational training, high = higher vocational training, university or PhD degree; ^b^ benefits : social security, unemployment benefits, disability allowances and other; ^c^ assessed at children’s age of 6 ; ^d^ assessed at children’s age of 10. | | | |

| **Table S2** Correlations between family SEP indicators (n=3796) | | | | | | | |
| --- | --- | --- | --- | --- | --- | --- | --- |
|  | Maternal education | Paternal education | Maternal employment | Paternal employment | Income | Benefits | Single parenting |
| Maternal education | 1^**^ | 0.49^**^ | 0.25^**^ | 0.10^**^ | 0.33^**^ | 0.18^**^ | 0.12^**^ |
| Paternal education | - | 1 | 0.12^**^ | 0.14^**^ | 0.34^**^ | 0.17^**^ | 0.18^**^ |
| Maternal employment | - | - | 1 | 0.15^**^ | 0.29^**^ | 0.25^**^ | 0.08^**^ |
| Paternal employment | - | - | - | 1 | 0.28^**^ | 0.24^**^ | 0.16^**^ |
| Income | - | - | - | - | 1 | 0.43^**^ | 0.54^**^ |
| Benefits | - | - | - | - | - | 1 | 0.29^**^ |
| Single parenting | - | - | - | - | - | - | 1 |
| ** correlation is significant at the 0.01 level (2-tailed); The table is based on the imputed dataset. | | | | | | | |

| **Table S3** Test for multicollinearity in model 2^1^ based on original data | | | | |
| --- | --- | --- | --- | --- |
|  | |  | Collinearity Statistics | |
| Covariates | | | Tolerance | VIF |
| *SEP indicators* | | |  |  |
|  | Maternal education | | 0.781 | 1.281 |
|  | Paternal education | | 0.770 | 1.299 |
|  | Maternal unemployment | | 0.923 | 1.084 |
|  | Paternal unemployment | | 0.951 | 1.051 |
|  | Household income | | 0.701 | 1.426 |
|  | Receiving benefits | | 0.933 | 1.072 |
|  | Single parenting | | 0.797 | 1.255 |
|  |  | |  |  |
| *Covariates* | | |  |  |
|  | Caries experience | | 0.958 | 1.044 |
|  | IOTN-DHC | | 0.718 | 1.392 |
|  | IOTN-AC | | 0.717 | 1.395 |
|  | Self-perceived orthodontic need | | 0.832 | 1.203 |
|  | Gender | | 0.985 | 1.016 |
|  | Age | | 0.990 | 1.010 |
|  | Ethnicity | | 0.918 | 1.090 |
| ^1^Model 2 investigates the Associations of all socioeconomic indicators with OHRQOL at children’s age of 10 | | | | |

| **Table S4** Associations of socioeconomic indicators with OHRQOL at children’s age of 10 | | | | | | | | | |
| --- | --- | --- | --- | --- | --- | --- | --- | --- | --- |
|  | Basic model | Crude model | +maternal  education level | +paternal education level | +maternal employment status | +paternal employment status | +household income | +receiving benefits | +family composition |
| Maternal education |  |  |  |  |  |  |  |  |  |
| Low | **-0.31**  **[-0.54 - -0.08]** | **-0.34**  **[-0.56 - -0.11]** | - | -0.08  [-0.34 - 0.17] | -0.21  [-0.44 - 0.02] | -0.18  [-0.41 - 0.04] | -0.14  [-0.37 - 0.10] | -0.18  [-0.42 - 0.05] | -0.19  [-0.41 - 0.04] |
| Paternal education |  |  |  |  |  |  |  |  |  |
| Low | **-0.44**  **[-0.51 - -0.38]** | **-0.45**  **[-0.68 - -0.22]** | **-0.30**  **[-0.56 - -0.05]** | - | **-0.33**  **[-0.56 - -0.10]** | **-0.31**  **[-0.54 - -0.08]** | -0.26  [-0.50 - -0.03] | **-0.30**  **[-0.54 - -0.07]** | **-0.30**  **[-0.53 - -0.07]** |
| Maternal employ |  |  |  |  |  |  |  |  |  |
| No paid job | **-0.24**  **[-0.33 - -0.16]** | -0.24  [-0.54 - 0.06] | -0.05  [-0.35 - 0.25] | -0.06  [-0.36 - 0.24] | - | -0.04  [-0.34 - 0.27] | -0.01  [-0.31 - 0.31] | -0.04  [-0.35 - 0.26] | -0.06  [-0.37 - 0.24] |
| Paternal employ |  |  |  |  |  |  |  |  |  |
| No paid job | **-0.81**  **[-0.99 - -0.63]** | **-0.81**  **[-1.39 - -0.22]** | -0.54  [-1.14 - 0.06] | -0.55  [-1.14 - 0.05] | -0.56  [-1.16 - 0.04] | - | -0.48  [-1.08 - 0.13] | -0.49  [-1.10 - 0.11] | -0.51  [-1.11 - 0.08] |
| Household income |  |  |  |  |  |  |  |  |  |
| No paid job | **-0.67**  **[-0.76 - -0.57]** | **-0.67**  **[-0.99 - -0.36]** | **-0.40**  **[-0.74 - -0.07]** | **-0.35**  **[-0.68 - -0.02]** | **-0.45**  **[-0.78 - -0.12]** | **-0.39**  **[-0.71 - -0.06]** | - | -0.30  [-0.65 - 0.05] | -0.33  [-0.70 - 0.04] |
| Receiving benefits |  |  |  |  |  |  |  |  |  |
| < 2000€ | **-0.68**  **[-0.80 - -0.56]** | **-0.68**  **[-1.07 - -0.30]** | **-0.47**  **[-0.85 - -0.08]** | **-0.44**  **[-0.83 - -0.06]** | **-0.52**  **[-0.91 - -0.13]** | **-0.45**  **[-0.83 - -0.06]** | -0.37  [-0.78 - 0.04] | - | **-0.43**  **[-0.82 - -0.03]** |
| Family composition |  |  |  |  |  |  |  |  |  |
| Single parent | **-0.53**  **[-0.63 - -0.44]** | **-0.54**  **[-0.87 - -0.22]** | **-0.36**  **[-0.68 - -0.03]** | **-0.33**  **[-0.65 - -0.01]** | **-0.37**  **[-0.69 - -0.05]** | **-0.33**  **[-0.64 - -0.01]** | -0.23  [-0.59 - 0.13] | **-0.43**  **[-0.82 - -0.03]** | - |
| The data are presented as linear regression coefficients (β) with 95%-confidence intervals (95%CI). The crude model is adjusted for gender, age and ethnicity only. Model 1 is additionally adjusted for confounders: caries experiences, orthodontic treatment need, aesthetic treatment need and self-perceived orthodontic treatment need. Model 2 is additionally adjusted for confounders and the other socioeconomic factors. Significant associations are printed bold. | | | | | | | | | |

| **Table S5** Associations of socioeconomic indicators with OHRQOL at children’s age of 10 | | |
| --- | --- | --- |
|  | Oral health related quality of life (OR [95% CI]) | |
|  | High | Low |
| n | 2007 | 1864 |
| Mean±SD | 51.22±2.91 | 46.44±2.91 |
| Maternal education |  |  |
| Crude | ref | 0.95 [0.83 - 1.09] |
| Model 1 | ref | 0.99 [0.87 - 1.15] |
| Model 2 | ref | 1.11 [0.94 - 1.31] |
| Paternal education |  |  |
| Crude | ref | **0.84 [0.74 - 0.97]** |
| Model 1 | ref | **0.86 [0.75 - 0.99]** |
| Model 2 | ref | 0.85 [0.72 - 1.01] |
| Maternal employment |  |  |
| Crude | ref | 0.97 [0.82 - 1.15] |
| Model 1 | ref | 1.01 [0.85 - 1.20] |
| Model 2 | ref | 1.06 [0.88 - 1.28] |
| Paternal employment |  |  |
| Crude | ref | 0.79 [0.60 - 1.04] |
| Model 1 | ref | 0.82 [0.61 - 1.08] |
| Model 2 | ref | 0.89 [0.66 - 1.22] |
| Household income |  |  |
| Crude | ref | 0.84 [0.71 - 0.99] |
| Model 1 | ref | 0.86 [0.72 - 1.03] |
| Model 2 | ref | 1.01 [0.80 - 1.30] |
| Receiving benefits |  |  |
| Crude | ref | 0.71 [0.57 - 0.87] |
| Model 1 | ref | 0.71 [0.57 - 0.88] |
| Model 2 | ref | **0.73 [0.57 - 0.93]** |
| Family composition |  |  |
| Crude | ref | 0.86 [0.72 - 1.04] |
| Model 1 | ref | 0.87 [0.72 - 1.04] |
| Model 2 | ref | 0.95 [0.76 - 1.19] |
| The data are presented as odds ratio’s (OR) with 95%-confidence intervals (95%CI). The crude model is adjusted for gender, age and ethnicity only. Model 1 is additionally adjusted for confounders: caries experiences, orthodontic treatment need, aesthetic treatment need and self-perceived orthodontic treatment need. Model 2 is additionally adjusted for confounders and the other socioeconomic factors. Significant associations are printed bold. | | |
